# Soil Metaproteomics for the Study of the Relationships Between Microorganisms and Plants: A Review of Extraction Protocols and Ecological Insights

**DOI:** 10.3390/ijms21228455

**Published:** 2020-11-11

**Authors:** Maria Tartaglia, Felipe Bastida, Rosaria Sciarrillo, Carmine Guarino

**Affiliations:** 1 Department of Science and Technology, University of Sannio, via de Sanctis snc, 82100 Benevento, Italy; mtartaglia@unisannio.it (M.T.); sciarrillo@unisannio.it (R.S.); 2 CEBAS-CSIC, Department of Soil and Water Conservation, Campus Universitario de Espinardo, 30100 Murcia, Spain; fbastida@cebas.csic.es

**Keywords:** soil metaproteomics, protein extraction, rhizosphere crosstalk, polluted soil

## Abstract

Soil is a complex matrix where biotic and abiotic components establish a still unclear network involving bacteria, fungi, archaea, protists, protozoa, and roots that are in constant communication with each other. Understanding these interactions has recently focused on metagenomics, metatranscriptomics and less on metaproteomics studies. Metaproteomic allows total extraction of intracellular and extracellular proteins from soil samples, providing a complete picture of the physiological and functional state of the “soil community”. The advancement of high-performance mass spectrometry technologies was more rapid than the development of ad hoc extraction techniques for soil proteins. The protein extraction from environmental samples is biased due to interfering substances and the lower amount of proteins in comparison to cell cultures. Soil sample preparation and extraction methodology are crucial steps to obtain high-quality resolution and yields of proteins. This review focuses on the several soil protein extraction protocols to date to highlight the methodological challenges and critical issues for the application of proteomics to soil samples. This review concludes that improvements in soil protein extraction, together with the employment of ad hoc metagenome database, may enhance the identification of proteins with low abundance or from non-dominant populations and increase our capacity to predict functional changes in soil.

## 1. Introduction

The term “rhizosphere” was coined in 1904 by the agronomist and plant physiologist Hiltner to identify the portion of soil near the roots that, as he first postulated, welcomed an abundant microbial community influenced by the root-released substances. Over the years, the definition of the rhizosphere has expanded and, to date, it is described as composed of the endorizosphere, the rhizoplane, and the ectorhizosphere [1,2]. The substances released from the roots greatly influence the rhizosphere microbial pool and guide the complex plant–microorganisms interactions. The amount and the composition of the root exudates depend on the plant species and the physiological state and can be altered by the environmental conditions and the biotic or abiotic stresses to which the plant is subjected [3]. The roots exude low- and high-molecular-weight organic compounds, mainly amino acids, peptides, proteins, monosaccharaides, disaccharides and polysaccharides, mucilage, phenolic compounds, secondary metabolites, and some important factors for microbial attraction such as malic acid and citric acid. Root exudates model the set of microorganisms present in the rhizosphere through species–species specific attraction/repulsion chemotaxis [4,5]. In fact, exudates can favor positive interactions such as symbiosis, mycorrhizae association, and recruitment of plant growth-promoting bacteria (PGPR) and can inhibit negative interactions such as herbivory, parasitism, competition, and allelopathy [1,6].

The rhizosphere of a polluted soil is generally deeply altered, as it has undergone particular and specific adaptations that are a consequence of the degree and type of pollution. The ecto–endophytic properties are altered and conditioned by polluting substances and by the soil stationary conditions (matrix composition and structure alteration, pH variation, etc.). Further complexity is added to the system by the engineering of the rhizosphere (PGPR, mycorrhizae, etc.) in bioremediation processes.

To understand the complex communication between plant and soil microorganisms, metagenomics and metatranscriptomics are not enough, and a metaproteomic approach must be used in order to assess the real functionality of soil microbes in the rhizosphere. In fact, metagenomics is subject to various limitations, such us the molecular extraction techniques, bioinformatics of annotation, and data processing, but above all for the lack of correlation between the presence of a functional gene and the actual activity of that gene [7]. Moreover, recent studies suggest that a great part of DNA in soil belongs to dead or dormant cells [8]. Even metatranscriptomics cannot provide us accurate information of gene functionality, considering the various post-transcriptional regulations to which mRNAs are subject. Hence, from a quantitative point of view, metagenomics and metatranscriptomics can give us a relative abundance of a functional gene and its mRNA, but not its effective functionality, intended as a protein expression [7,8,9]. The metaproteomics approach allows us to analyze the taxonomic data, can offer a functional characterization of the biomass present in the rhizosphere, and has great potential as it can help identify the metabolic dynamics between and within species [10,11,12,13,14]. Although the analysis of the total rhizosphere proteome can provide fundamental information to understand the environment–plant–microorganism interactions, soil protein extraction is very complex due to the nature of the soil matrix. The methodological challenges in protein extractions from soil are many; the matrix is complex, chemically and physically heterogeneous, and very variable. Moreover, to extract intracellular proteins, a passage of cell lysis is necessary. Furthermore, the low quantity of proteins present binds humic substances, making their extraction even more complex [15,16]. Metaproteomics analysis of polluted soils has great potential to shed light on the impact of diversity and microbial functionality established in a disturbed soil, assigning proteins to specific phylogenetic and functional groups. This aspect is amplified at the rhizosphere level where particular co-metabolic systems and relationships between different organisms persist, particularly in the dynamic prospects of pollutant degradation. In addition, the metaproteomic application becomes an important tool when the rhizosphere is engineered by adding bacterial and/or fungal cocktails, biostimulants, and nanomaterials to enhance the effect of bioremediation. In fact, the metaproteomic analysis would allow us to obtain a clear vision of the co-metabolic perspectives generated by the engineering of the rhizosphere and it would offer novel information on the genetic and metabolic processes occurring during soil bioremediation. However, soil proteins are associated with humic substances and clay particles [17,18,19], making it hard to obtain a sufficiently clean protein extract for further processing and downstream analyses. To overcome these challenges, sophisticated protocols for sample preparation must be tested. This review provides the reader with detailed information on the different protocols for protein extraction from soil samples, focusing on those interested in rhizosphere soil, offering a panorama that is at least exhaustive of the current extraction methodological trends and of the current quali–quantitative limits. It also intends to provide an element of discussion on the future prospects and the need and usefulness of metaproteomic studies on polluted rhizospheres and bio-mediated soils.

## 2. Methods: Selected Studies for This Review

The study of protein expression in environmental samples is a hot topic. On the Web of Science (http://www.webofknowledge.com/), using “soil” and “metaproteomics” as topics it was possible to find 126 records: 95 articles, 28 reviews, two proceedings papers, one book chapter (plus one data paper, one reprint, and one editorial material) published from 1990 to date (June 2020). According to their pertinence, 71 were selected. The same research on Scopus (https://www.scopus.com/) yielded 109 documents: 73 articles, 20 reviews, eight book chapters, three short surveys, two conference papers, one data paper, one letter, and one undefined (June 2020). Of the 73 articles, only those that were truly relevant and in English were taken into consideration. A cross-comparison between these 54 elected papers and the 71 obtained from the search on Web Of Science showed that 49 papers were shared between the searches, four were present in Scopus but not in Web Of Science and vice versa; 22 articles were present in Web Of Science but not in Scopus, with a final count of 75 articles taken into consideration. The same search on PubMed (https://pubmed.ncbi.nlm.nih.gov/) provided 83 results, 31 of which were rejected because they were reviews, non-related or non-English articles. Of the remaining articles, only five were not already present in the search results from the Web of Science and Scopus and were added to the total papers taken into consideration.

## 3. Soil Matrix Components that Interfere with Protein Extraction

Metaproteomics has great potential to provide new information on rhizospheric interactions, but the success of this approach depends on the quantity and quality of the extracted proteins. The soil, in fact, is one of the most complex matrices for the extraction of proteins due to its physical, structural, and biological complexity, its heterogeneity, and due to the presence of a mixture of organic macromolecules that interfere with the metaproteomic workflow [14,20]. Humic substances, co-extracted with proteins, interfere with the quantification of proteins with spectrophotometric techniques and appear as brown streaks on SDS-PAGE and 2D-PAGE gels, preventing the detection of protein bands/spots. They also cause peptide signal suppression in LC-MS/MS measurements [19,21,22,23]. The more or less strong bonds between proteins and the soil matrix are influenced by the specific characteristics of the soil under analysis, such as pH, composition, and mineral content [23,24,25,26]. Most extraction protocols involve the use of liquid phenol to remove humic substances [21,23,27,28,29] and polyvinylpolypyrrolidone (PVPP) [11,23]. However, it is necessary to take into account the limited safety in the use of phenol, and the poor results obtained with PVPP. In order to remove, or in any case limit, the interference of humic acids, various strategies have been tested such as the precipitation of humic substance with trivalent aluminum ions, the use of salt solution of inorganic divalent or trivalent cations to desorb the immobilized proteins from humic substance, and the addition of CaCl_2_ [19,30,31]. However, the fact that many proteins are linked to humic substances is functional to their stability. For instance, many extracellular enzymes are known to be stabilized within humic substances [32]. Therefore, any method that attempts to remove humics will likely discard proteins as well and interfere with their biological significance and function. In addition, it is difficult to cleave proteins from humics. Another interfering substance is keratin that is not a problem until its concentration remains low compared to the proteins of interest. If its concentration, on the other hand, is too high, it masks the signal of the peptides of interest and shows artifacts mainly at 54–57 kDa and 65–68 kDa [33,34]. To minimize keratin contamination, all materials should be handled with nitrile gloves, and latex must be avoided because it contains significant quantities.

## 4. Soil Protein Extraction

**Figure 1:**
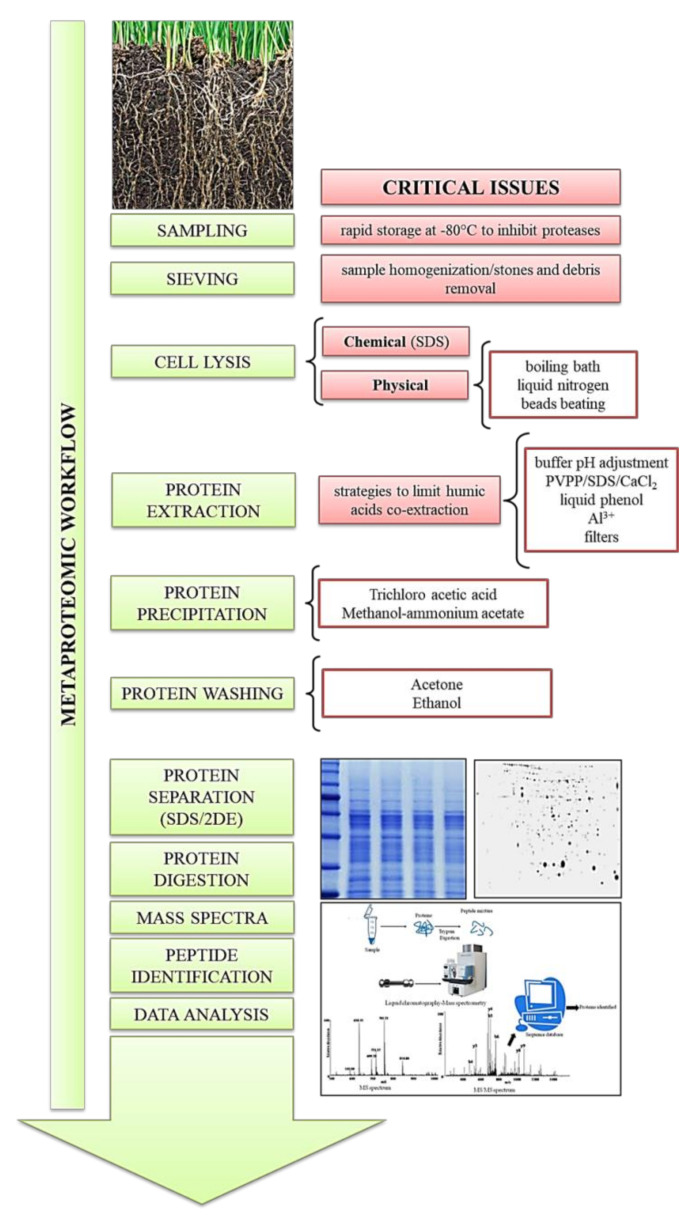
Metaproteomic workflow and critical issues.

Different approaches and protocols have been used over the years and are present in literature in order to obtain feasible protein extraction in quantitative and qualitative terms (Supplementary Materials Table S1). The general workflow is almost common to all protocols and involves sample collection and storage, cell lysis, protein extraction, and a series of washes (Figure 1). The protein extract thus obtained is ready for quantification, SDS page or 2DE electrophoresis, and mass spectrometry (MS) [15,35]. Protein extraction, obviously, begins with the collection of the soil sample that is sifted to remove stones and debris and make the sample homogeneous. To inactivate the proteases present in the soil, the sample must be stored at −80 °C until use. To extract both extracellular and intracellular proteins, a first cell lysis step is necessary. In a first work that recognized the importance of protein extraction from environmental samples, Ogunseitan [36] tested two extraction techniques. Both methods provided as a first step a sample wash with ice-cold 50 mM Tris-HCl, pH 7.4, followed by two types of physical cell lysis. With the “boiling method”, cell lysis was obtained by placing the samples in a boiling water bath for 10 min in 1:1 water and extraction buffer. In the “freeze–thaw method”, instead, hot–cold cycles were used in order to free intracellular proteins before the extraction. A series of centrifugations and the use of a filter to remove debris and DNA allowed protein separation by electrophoresis. The proteins extracted with both methods were between 14 kDa and 97 kDa. In quantitative terms, on the other hand, with the first method, the estimated protein concentration was 1–5 µg/g, and with the freeze–thaw method, 20–50 µg protein/g [36]. Murase et al. [37] restricted the pool of interest to extracellular proteins only and therefore developed a protein extraction from soil that did not include cell lysis. Extracellular proteins, derived from the death and disruption of cells of organisms, or as extracellular enzymes that are continuously excreted by microorganisms and plants, are present in very small quantities. For this reason, the extraction was carried out on a starting sample of 100 g. The work showed how the buffer pH affects the extraction quality; in fact, a higher pH of the phosphate buffer resulted in a greater quantity of soil organic matter (SOM) included in the prepared sample, resulting in a high background with unclear protein bands on SDS—PAGE [37]. The protocol proposed by Ogunseitan [36] was taken up and modified in a subsequent work in which the protein extraction was carried out on soil contaminated with cadmium. The protocol and the extraction (with a protease inhibitor cocktail to prevent proteolysis) have been optimized to obtain a better quantitative yield of the extraction. Cell lysis was obtained with two different techniques: the first involved four repeated cycles of freezing in liquid nitrogen and thawing at 25 °C. The “bead beating method” instead, used 0.2 g of glass beads (Sigma, 150–212 m in diameter) per gram of soil stirred in a Fast Prep machine. The results obtained from the subsequent protein quantization showed that 32% more protein was extracted when using the snap freeze method when compared with the amount of protein obtained from the bead beating one [38]. In these early papers, SDS-PAGE showed proteins between 90 and 110 kDa, and few between 36 and 51 kDa. Schulze et al. [39] utilized MS-based proteomics to analyze proteins isolated from dissolved organic matter (DOM) using suction plates installed at different depths in a deciduous forest. In this procedure, only extracellular proteins were separated from the inorganic material and were separated from the organic molecules by gel filtration and subject to SDS-PAGE. After digestion with trypsin, the peptides obtained were separated with Nanoflow Liquid Chromatography (LC) before analysis with tandem mass spectrometry (MS/MS). This made it possible to identify the type, functions, and origin of the extracellular proteins extracted; furthermore, sampling at different depths made it possible to establish that there was a progressive reduction in the number of proteins identified as the soil depth increases [39]. Subsequently, an extraction method was developed in which an incubation with NaOH can break the microbial cells and separate the proteins linked to minerals and humic acids, while the consequent extraction, a classic protein extraction from plant material [40], involves the use of phenol to separate proteins from the humic organic matter. At the same time, phenol allows the removal of DNA and RNA that negatively influence the subsequent separation of proteins on gel [41]. However, with this extraction technique, it was not possible to completely eliminate the humic substance that appeared as a brown streak on the polyacrylamide gel of the SDS-PAGE [21]. To focus on the problem of the close interaction between proteins and humic acids, a study was done to test the efficiency of three different extractants, 0.1 M sodium pyrophosphate (pH 7), 67 mM phosphate buffer (pH 6), and 0.5 M potassium sulphate (pH 6.6), in recovering total protein quantity and the β-glucosidase enzyme from two natural forest soils [42]. The extracellular proteins were extracted with sodium pyrophosphate at a 1:5 *w*/*v* ratio, at 37 °C for 24 h under shaking, according to a previous protocol by the same authors [43] and with phosphate buffer and K_2_SO_4_ at a 1:3 *w*/*v* ratio at room temperature for 1 h under shaking, modifying a previous protocol [37]. The total proteins instead, were extracted by the chloroform fumigation–extraction method [44]. Despite that polyvinylpyrrolidone (PVPP) and a filtration column were used, the colorimetric assay to establish protein concentration was difficult due to the presence of co-extracted interfering substances as already found in previous studies [30]. The SDS-PAGE showed a high intracellular protein concentration using sodium pyrophosphate, while a higher concentration of extracellular protein was shown using potassium sulphate as an extractant. However, the need to develop protocols with better purification phases was still evident [42]. Chen et al. [45] devised a sequential extraction method, through sequentially extracting soil in 0.05 M citrate, pH 8, or citrate plus SDS buffer (1% *w*/*v* SDS, 0.1 M Tris-HCl, pH 6.8, 20 mM dithiothreitol (DTT)) at room temperature, followed by a classic phenol extraction and a proteic precipitation with five volumes of cold methanol plus 0.1 M ammonium acetate overnight at −20 °C. DTT, not used in previous protocols, was useful for reducing intra-molecular and inter-molecular disulphide bonds between cysteine residues by helping protein solubilization and aggregate disruption. The protein pellet obtained after centrifugation was washed with cold methanol and twice with cold acetone, air-dried, and solubilized for SDS-PAGE and 2DE. Although the results showed that the SDS buffer was more powerful than other extractants tested and phenol extraction was effective to remove interfering substances, this approach was not considered applicable for deep proteome studies, due to the reduced number of proteins extracted and to the low resolution of 2-DE separation [45]. A new protocol for deep proteome characterization of microorganisms in soil was developed by Chourey et al. [22]. This protocol involves the use of an alkaline-SDS buffer with a high concentration of DTT, followed by a 10 min boiling bath in SDS buffer. The combination of a detergent-rich buffer and thermal action induced cell lysis and the inhibition of protease activity. Cell lysis was followed by the precipitation of proteins with the addition of trichloroacetic acid (TCA) overnight at −10 °C. The protein pellet obtained was then subjected to a series of cold acetone washes. The pellet was then solubilized in a guanidine buffer prior to liquid chromatography-mass spectrometric characterization [22]. This approach tested on soils inoculated with bacteria identified 500–1000 proteins by 2D-LC–MS/MS. This protocol was well described in detail in later work [46]. The same procedure [22] has been utilized in different subsequent studies by Bastida et al. [47,48,49]. The combined protocol of Chen et al. [45] was refined and modified in a later work by Wang et al. [50], increasing the extracted protein content and shortening the extraction time, replacing the sequential citrate and SDS buffer extractions with a single treatment of the two buffers. Through this modified protocol, an average of 1000 spots on a 2-D gel of rice soil samples was detected, of which 122 proteins were identified by MALDI-TOF/TOF–MS [50]. Keiblinger et al. [23] compared four different protein extraction procedures: SDS extraction without phenol, NaOH in combination with phenol extraction, SDS buffer followed by phenol extraction, and the combination of SDS/phenol with prior washing steps. Proteins were analyzed by two-dimensional liquid chromatography/tandem mass spectrometry (2D-LCeMS/MS) and, in both soil types, extraction with the SDS buffer followed by phenol resulted in an increase in the numbers of extracted proteins [23]. Lin et al. [51] adopted the protocol of Wang et al. [50], testing it to obtain a metaproteomic profile of sugarcane rhizosphere soil. The combination of the extractions with the citrate buffer and the SDS buffer, followed by phenolic extraction, allowed the resolution of 143 protein spots with high resolution and repeatability, 109 of which were successfully analyzed by MALDI TOF-TOF MS [51].

In all the protocols seen so far, the main obstacle to performing good soil protein extraction seems to be the co-extraction of humic acids, which are arduous to separate from proteins and make it difficult, if not impossible, to quantify proteins with spectrophotometric approaches and to obtain a good resolution on polyacrylamide gels, for both the SDS-PAGE and 2DE. In fact, soils with high microbial biomass are usually associated with a higher organic matter content (including humics). In contrast, soils with low organic matter usually also have lower microbial biomass, which also limits protein extraction. Electrostatic attraction drives encapsulation of positively charged proteins, at pH 5 to 8, by soil humic and fulvic acids derived from terrestrial and mixed terrestrial–aquatic sources [52]. To obtain a complete soil metaproteomic analysis, it is necessary to directly extract both extracellular and intracellular proteins, and therefore, as already mentioned, the passage of cell lysis prior to protein extraction is fundamental. However, the disadvantages of the direct extraction method includes incomplete lysis and co-extraction of humic substances [14,45]. In order to overcome these issues, Nicora et al. [53] tried a pre-treatment of the soil sample with an amino acid mixture to evaluate the ability to produce a non-adsorptive surface on soil samples. Furthermore, the compatibility of this procedure with the subsequent protein extraction, tryptic digestion, and mass spectrometric analysis was assessed. The results showed the possibility of increasing protein identifications through blockage of binding sites for a variety of soil and sediment textures using proteins from lysed *Escherichia coli* cells, although the need for a better extraction buffer was still underlined [53]. Given the heterogeneity of the proposed methods, three different protocols [21,22,38], were tested to compare their effectiveness in cell lysis, humic acid cleaning, and protein purification [47]. Although the quantity of extracted proteins is limited with all the protocols tested, the comparison showed that the extraction technique greatly influences the type of extracted proteins and consequently the taxonomic and functional information obtained from them. Importantly, this study quantified the yield of protein extraction through the analyses of amino acids and not through colorimetric approaches, thus avoiding interferences related to humic substances. In fact, Chourey’s method allowed a greater extraction and identification of different proteins, especially bacterial, while Singleton’s method allowed a greater extraction of fungal proteins [47]. However, the need for a robust analysis of the different extraction protocols for soils with different characteristics was still evident. The study of all proteins recovered directly from environmental samples must take into account the high soil heterogeneity that makes the possibility to develop a universal extraction protocol suited for all different matrix types difficult. Mattarozzi et al. [23] tested three different protocols in a metaproteomics study based on liquid chromatography-high resolution mass spectrometry (LCHRMS) in serpentine soil contaminated by heavy metals, mainly Ni, Co, and Cr. Three different protocols were applied to the homogenized soil samples: the first involved the use of the NoviPure Soil Protein Kit (Qiagen), the second was in accordance with Chourey et al. [22], and the third involved phenol-SDS extraction [23]. The comparison between these methods established that the protocol proposed by Chourey was particularly efficient for protein extraction, allowing a greater extraction of membrane proteins. The NoviPure Soil Protein Kit instead, probably thanks to the strong mechanical action in lysing the cells, allowed a greater extraction of intracellular proteins. The phenol-SDS method allowed extracting proteins equally distributed in the intracellular and membrane compartment. In quantitative terms, on the other hand, the extraction with the NoviPure Soil Protein Kit yielded the lowest values for the colorimetric assay. Given the higher number of unique identified proteins obtained by mixing the extracts of the three methods, [54], it is recommended that different extraction approaches be combined to improve metaproteomic analysis. Liu et al. [55] applied the SDS–phenol method with a previous treatment of forest soil samples ground in liquid N_2_. The subsequent analysis of the protein pellet obtained allowed the detection of 640 proteins by nano-LC-MS/MS [55]. In improving the extraction protocol, Qian and Hettich [56] took into consideration the insoluble nature of humic acids in acid solution, and the greater molecular weight of humic acids compared to peptides. The co-extract of humic substances, which interferes with the correct protein extraction from soil samples, is treated with the combination of a detergent and precipitation in trichloroacetic acid (TCA) and a filtration phase at pH 2~3 with a 10-filter kDa. According to the authors, this approach should improve extraction in qualitative terms, without adversely affecting it in quantitative terms and without interfering with subsequent analyses [18]. An experiment was conducted to identify the best protein extraction approach for the extracellular metaproteome that is diluted in soil samples. Various methods for protein concentration, extraction, precipitation, and solubilization have been tested; however, most methods failed to provide pure samples and therefore negatively influenced protein migration on gels and gel background clarity. The combination of phenol-based and TCA-acetone methods has been proposed as a strategy to capture the entire extracellular proteome [56]. The main limiting factor of this approach is the cultured growth of the microbial material prior to protein extraction. This strategy, which certainly increases the quantity and quality of the extracted protein sample, is focused only on the microbial communities and ignores all the extracellular proteins present in the rhizosphere due to the interaction with the roots and their exudates. Despite the progress made, protein extraction needs to be improved in order to efficiently characterize microbial functions in soil. Since soil characteristics, such as organic C, clay, and humic acid contents, represent the main factors that hinder good protein extraction, especially for extracellular proteins, in a recent work, the efficiency of two different extracellular protein extraction methods ofr different soils, without detergents or ultra-sonication, was compared [57]. The first one with a pH 6.5 buffer [58] and the second with an extraction protocol, in accordance with previous works [26,59] based on 0.1 M sodium pyrophosphate buffer, at pH 7.1. The work showed that the type of buffer not only influenced the quantity, but also the type of extracted protein. The absence of a standardized method for the extraction of soluble proteins from the soil has been discussed in a recent work, in which the capacities of the different extractors was tested on three soil types (Cambisol, Ferralsol, and Histosol) [60]. To evaluate the effective yield of these protocols, soluble 14C-labeled proteins were added to the soil samples. For protein extraction, deionized water, Na-pyrophosphate (0.01, 0.05, 0.1 M; pH 7.0), Na-citrate (0.01, 0.05, 0.1, 0.5 M; pH 8.0), Tris-SDS (0.01, 0.05, 0.1 M SDS with 0.05 M Tris; pH 7.0), phosphate buffer K (0.01, 0.05, 0.1, 0.5 M; pH 6.0 and 8.0), CaCl_2_, NaOH and K_2_SO_4_ (0.01, 0.05, 0.1, 0.5 M), methanol, and ethanol (25%, 50%, 75%, 100% *v*/*v*) were used. The results obtained by Greenfield and colleagues have shown that protein extraction is strongly influenced by solvent and soil characteristics. Although no type of extraction permitted complete incubated protein extraction, the maximum recovery was observed with NaOH (0.1 M; 61–80%) and Na-pyrophosphate (0.05 M, pH 7.0; recovery 45–75%) [60]. However, this study focused only on hydrophilic proteins, and is therefore not conclusive for total protein extraction from this type of matrix. In a 2018 study by Callister et al. [61] an extraction protocol from human tissue [61] was modified and adapted for soil samples. To extract a good quantity of protein, 60 g of silt-loam soil was used. Cell lysis was entrusted to the mechanical action of a mix of beads (0.9–2.0 mm stainless steel beads, 0.1 mm zirconia beads, and 0.1 mm garnet beads). Protein extraction occurred using 2:1 chloroform: methanol (*v*/*v*) in a 5:1 ratio over sample volume in MilliQ water, with vigorous mixing. After sonication, incubation at −80 °C and centrifugation, the protein-containing interphase was taken and precipitated in ice-cold 100% methanol. The pellet obtained after centrifuging was frozen and lyophilized before being resolubilized in an SDS-Tris buffer containing DTT, then incubated for 30 min at 300 rpm, 50 °C. The samples were centrifuged and, in order to remove humic substances, the protein extracts were again precipitated in 20% trichloroacetic acid (TCA); the obtained pellet after the centrifugation underwent three washes in ice-cold acetone [62]. Although the protein pellet obtained was suitable for 1D LC-MS/MS and 2D LC-MS/MS with a good depth of coverage, the reproducibility was poor; moreover, the protocol is particularly laborious and requires more time than the others proposed. Protein sample preparation for mass spectrometry is a key step for a good metaproteomics approach; in gel-free metaproteomic workflows, protein purification is critical and starts with a precipitation stage. Precipitation in TCA is the most used and induces the formation of stable molten protein globules, which are difficult to resolve in aqueous buffer for proteolytic digestion before the MS analysis. To overcome this problem Eddhif and colleagues proposed a washing step of TCA-precipitated proteins in ethanol/HCl. This approach, tested on several matrices including soil, allowed an increase in protein recovery in aqueous buffer from 50 to 96% [63,64].

While a limited number of protocols are currently available for the extraction and purification of soil proteins, several protocols for the isolation of soil DNA are available. In these works, the addition of Al^3+^ was proposed and the pH adjustment of the extraction solution to 5.5-6 to effectively remove humic substances [34,65,66,67,68]. This soil DNA extraction protocol has been adapted for protein extraction from soil [19], evaluating the most relevant parameters of the protocol, such as buffer pH and detergent concentration, to obtain the maximum recovery of proteins and minimum levels of humic substance co-extraction. To evaluate the performance of this approach, several soil samples with variable physico–chemical characteristics were subjected to the classic extraction with phenol, the NoviPure Soil Protein Kit, and the adapted protocol by Mandalakis et al. [19], that provided the flocculation of humic substances with trivalent aluminum ions. The latter has proven to be a non-toxic, inexpensive, and viable approach for the purification of proteins from soils [19]. In one of the most recent works, an optimization of already cited protocols [21,23], with modifications at several steps, was done to improve the metaproteomic analysis of rhizospheric samples [69]. The use of polyvinylpolypyrrolidone (PVPP) to clean samples from humic acids and liquid nitrogen to minimize proteolysis was proposed again in this protocol. The proteins were extracted with a double extraction, first with 10% TCA/acetone and then with phenol (pH 8.0) and SDS buffer. Although the mass spectrometry proteomics data showed a total of 696 proteins, the images on the SDS-PAGE showed a poor quantity and quality of the bands obtained [69].

### 4.1. Kit for Protein Extraction from Soil

Given the potential of the proteomics approach in the analysis of environmental sample functional status and the various critical issues during the protein extraction from complex matrices, numerous protein extraction kits have been developed and are now available. Since 2013, a kit for soil protein extraction has been available (NoviPure^®^ by MO BIO Laboratories, Carlsbad, CA, USA); although this kit does not involve toxic reagents and its use is practical and fast, it is rather expensive and does not allow adapting the extractive protocol to the specific characteristics of the soil under consideration. Despite the limitations, protein extraction is entrusted to the NoviPure Soil Protein Kit only, while in some papers the kit performances are compared to classic extraction techniques (Table 1). From the comparison, the use of the kit seems to provide comparable results, if not inferior in quantitative and qualitative terms, compared to traditional extraction techniques [19,54,70,71,72].

### 4.2. Co-Extraction for Multiomics Approach

In order to obtain significant data from the “omics” approaches, DNA, RNA, proteins, and/or metabolites must be extracted simultaneously from the same sample with a co-extraction protocol that allows the extraction of all the biomolecules of interest without altering them [79]. The method is based on the co-extraction of DNA, RNA, and proteins [80], which provides for the use of phenol-chloroform, which has been taken up and adapted in various works to allow the co-extraction from soil samples with sufficient yields to allow downstream analysis [81,82,83]. Given the potential of the multiomic approach for soil samples, to reduce time and costs, Nicora et al. [84] proposed a 3-in-1 method for the simultaneous extraction of metabolites, proteins, and lipids (MPLEx) protocol [84]. The protocol, previously used only for lipid extraction, involves the use of organic solvents, chloroform, methanol, and water. Chloroform is not miscible with water and allows the three-phase chemical separation of the components: the aqueous phase contains the hydrophilic metabolites, the interface the proteins, and in the lower chloroform phase the lipid layer. The MPLEx protocol can be used on most soils; however, in highly organic soils such as peat, soil debris remains in the intermediate layer, negatively affecting protein extraction [84].

## 5. Metaproteomics Applications

**Figure 2:**
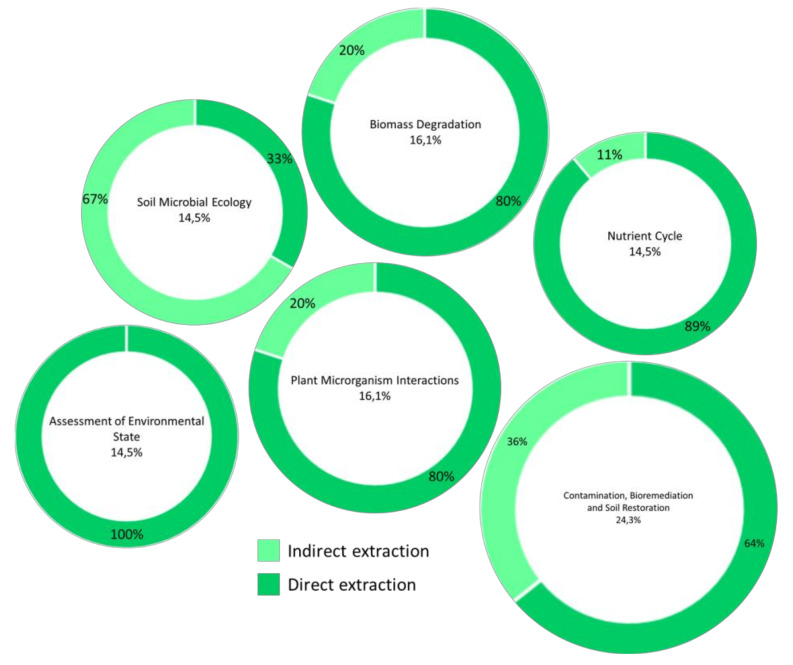
Percentage of metaproteomics applications out of the total number of works taken into consideration with frequency of direct and indirect approaches in extraction.

Among many other factors, root exudates modulate rhizosphere microbial activity, actively take part in the geochemical and nutrient cycles in the soil, and in the decomposition of organic matter and mineralization processes, affecting the plant growth and health status. Furthermore, given the ability of some microorganisms to biodegrade or detoxify polluting substances accumulated in soils, they play a fundamental role in soil bioremediation, and metaproteomics can reveal new pathways and populations involved in bioremediation. Most of these bacteria, very active in the soil, are not culturable, but vary greatly in quantity and functionality in the presence of host plants. Given the urgent need to clean up soils polluted by human activities, the search for effective and easily applicable strategies is a topic of great interest. Consequently, the metaproteomics approach, with protein extraction directly from rhizospheric soil samples, has wide potential applications (Figure 2; Table 2), being able to provide not only taxonomic but also functional information, providing a complete picture of functional–taxonomic relationships in soil in a given time [85].

In this review, we focused on metaproteomics applications related to the interactions between plants and microorganisms in the rhizosphere, and the presence of contaminants and bioremediation processes.

### 5.1. Plant–Microorganism Interactions

Soil rhizospheric metaproteomics is a promising tool to understand the complex plant–microorganism interactions and the rhizosphere in situ biological/functional properties and how they can adapt to environmental factors. In a metaproteomics study on rice crop phyllosphere and rhizosphere, Knief et al. [95] used the All Prep DNA/RNA/Protein MiniKit (Qiagen) and identified 4600 total proteins. The comparison among differentially expressed proteins between the rhizosphere and phyllosphere showed that those involved in nitrogen fixation and methanol use processes were particularly abundant in the rhizosphere sample [117]. In the rice rhizosphere, Wu et al. [117] identified 122 proteins, mainly involved in energy and secondary metabolism, protein and nucleotide biosynthesis, signal transduction, and resistance. In order to evaluate how the consecutive *Rehmannia glutinosa* monoculture alters the rhizosphere, the results of the metaproteomics analysis of rhizosphere samples taken in subsequent years were compared. Of the 152 spots obtained by 2-DE, only 49 proteins were identified by LIFT-MALDI TOF-TOF-MS, and of these, 33 were differentially expressed. According to the KEGG (Kyoto Encyclopedia of Genes and Genomes) database, these were mainly involved in primary metabolism, in secondary metabolism, and in the response to stress [117]. The metaproteomics approach was used in a later work by Lin et al. [51] to evaluate the effect of yield decline in ratoon sugarcane. Of the 109 protein spots identified by LC-MALDI TOF/TOF, only 33 were differentially expressed. Of these proteins, those derived from plants were mainly involved in primary metabolism and stress responses, while microbial ones were mainly involved in membrane transport and signal transduction [51]. Bao et al. [88] combined a metaproteomic study with catalyzed reporter deposition–fluorescence in situ hybridization (CARD-FISH) to analyze methanotrophs localized in rice root tissues. Further, the metaproteomics approach has been used on a truffle-ground soil already analyzed from a metagenomics point of view [88]. These authors identified proteins from fungi, bacteria, and Viridiplantae and analyzed their function according to the KEGG database [88]. Microbial activities in the rhizosphere affect plant health; consequently, the composition and functions of the soil biome are fundamental for the development and engineering of sustainable agriculture. Manikandan et al. [104] compared protein expression in wilt-infected and healthy tomato rhizospheres. The protein extracts were identified and were derived from plants, bacteria, and fungi. Among these, 11 proteins were differentially expressed and were involved in plant defense responses [104]. Starke et al., [107], with a combination of genomic and proteomics approaches, identified the bacteria involved in the short-term assimilation and tracked the flow of plant-derived N. Interestingly, this study provides one of the first insights into the combined use of protein and stable isotope probing (SIP) in soil rhizosphere to reveal the involvement of microbial populations and functional pathways in relation to the nitrogen cycle [107]. Moreover, in order to identify the repertoire of secreted microbial proteins and use to cooperate or compete and gain information about the interaction pathways between roots and rhizosphere communities, Bona et al. [89] focused attention on the metaproteomics analysis of the rhizosphere of *Vitis vinifera* cv. Pinot Noir. The extracted protein analysis allowed the identification of 150 bacterial genera and the understanding of the functional state of the rhizosphere [89]. In 2019, the first metaproteomics study on the maize rhizosphere was reported and identified 696 proteins with different functions from 393 different species. Most of these proteins belonged to three functional groups: carbon metabolism, glycine biosynthesis, and IMP (Inosine Mono Phosphate) biosynthesis [69].

### 5.2. Rhizosphere Under Environmental Factors

Several works in literature aimed to elucidate how the natural environmental conditions or their alterations influence and alter the rhizosphere. The long-term effects of deforestation on soil microbial communities have been explored through protein extraction from soil, carried out according to [22], allowing the identification of 715 proteins in a deforested soil sample and 472 from a forest soil sample [89]. The taxonomic and functional analyses allowed the conclusion that if on the one hand deforestation causes a loss of total microbial biomass; on the other, it induces an increase in diversity [89]. In a subsequent work by Liu et al. [55], a temperature rise of +4 °C in a sample of forest soil was applied in order to quantify the effects of global warming on the rhizosphere. The analysis of the extracted proteins allowed the establishment that the bacterial proteins far exceeded those of a fungal nature, and that the differences after the exposure to the increase in temperature were few in taxonomic terms, confirming the ability to adapt and microbial resilience, while from a functional point of view, there was a marked increase in the proteins involved in soil respiration. Confirming the crucial importance of climatic conditions in determining the abundance of soil microbial proteins and the metabolic activities of the main phyla present there, Bastida and colleagues assessed the metaproteome of soils from different climatic zones associated with distinct vegetation patterns and under an increasing temperature [123,124]. The results showed that the increase in temperature corresponded to an increase in the proteins derived from Actinobacteria and Planctomycetes, and decrease of those of Proteobacteria. From a functional point of view, on the other hand, all proteins related to cellular energetic processes and soil respiration were over-expressed [123]. The protocol proposed by Chourey et al. [22], suitable for semiarid soils, was used to evaluate the effects of water/salinity stress on the soil microbial community of a grapefruit agro-ecosystem model. The metaproteomics approach made it possible to identify functional changes and adaptations to stress in the pool of microorganisms present in the rhizosphere whose phylogenetic diversity remained constant [109].

### 5.3. Contaminants, Bioremediation, and Soil Restoration

The possibility of using soil proteomic analysis as an indicator of cadmium contamination was proposed by Singleton and colleagues in a 2003 paper that highlighted the reduction in the number of total proteins extracted from soil samples containing cadmium. However, no subsequent analysis was performed on the protein extracts obtained [38]. Benndorf and colleagues, modifying their own previews of protein extraction protocols, analyzed the metaproteome of soil’s anoxic microbial communities and their ability to biodegrade petroleum hydrocarbons, especially benzene that is particularly recalcitrant in the absence of oxygen. Through the separation of cells from soil granules, cell lysis by ultrasonication, and purification of proteins by phenol extraction, 240 protein spots on 2D-gels were found, and several proteins were successfully identified. These included some enzymes, such as enoyl-CoA hydratase, involved in the anoxic degradation of xenobiotics [92]. Combining different extraction techniques, as previously seen, the metabolic and functional effects in metal-tolerant and metal hyperaccumulator plants, growing in soil contaminated by heavy metals (Ni, Co, Cr), were analyzed [54]. Of the 800 proteins identified, most were involved in stress response or metal uptake, highlighting the ability of the rhizosphere to plastically adapt to microenvironment conditions [54]. Christensen et al., combined metaproteomics and metagenomics to evaluate the presence, distribution, and diversity of Hg-methylating bacteria, responsible for the microbial conversion in the soil of methyl mercury (MeHg), a toxic contaminant [106]. Chourey et al. [100], using an improvement of their previously published protocol validated the usefulness of the metaproteomics approach, identifying the main microbial communities and the pathways activated following contamination by uranium and nitrate. Metagenomics and metaproteomics approaches were applied by Guazzaroni and colleagues to reconstruct the degradation pathway of naphthalene. The results of the multiomics approach indicated the biodegradation capacity of microbial communities, which are extremely suitable for naphthalene. Furthermore, the bio-stimulation of polluted mesocosms induced the release of recalcitrant compounds from the soils that seemed to remodel the composition in terms of the variety and abundance of the soil microbial community [110]. In a recent work, Chen et al. [97] analyzed the effect of dibutyl phthalate on the microbiome of agricultural soils, demonstrating through metabolomics, metaproteomics, and enzyme activity assays, the presence of this compound in the soil induces an up-regulation of a series of microbial proteins involved in the metabolism and transport of sugars. Consequently, there was a clear decrease in glucose in the soil that could lead to negative effects on the rhizosphere [97]. In a 2016 paper, Bastida et al. [48] evaluated the bioremediation effect of compost amendment in hydrocarbon-contaminated soils. Metaproteomics showed that the addition of compost, inducing an increase in microbial catabolic activity, promoted a net reduction of polycyclic aromatic hydrocarbons [48]. The same authors pointed out the correlation between the organic amendment and phyla-lifestyles, as well as the main biochemical degradation pathways in this polluted soil. By combining the metagenomics and metaproteomics approaches, the biocatalytic potential of the microbial community in a soil contaminated with used cooking oil was evaluated, confirming its lipolytic activity [122].

## 6. Conclusions

Soil metaproteomics is a research field of great interest given the information obtained from it, not only to understand the physiological and functional states of the interaction between microorganisms and plants present in the rhizosphere, but also to be able to “engineer” the rhizosphere, modifying the microbial pool and/or resident plants, in response to environmental stress, climate change, and in order to promote the bioremediation of contaminated soils. Indeed, more than any other approach, the analysis of the rhizosphere metaproteome allows us to evaluate the responses of the rhizosphere microbiome to environmental changes and also to determine how the rhizosphere can respond to targeted engineering interventions to enhance its capabilities. To evaluate the phylogenetic and functional correlations of a contaminated soil, the proteomics approach represents a valid means to test the effective potential of different bioremediation and soil restoration approaches. In spite of the limitations of metaproteomics, which are certainly shared by many other soil approaches based on the extraction of biomarkers in soil (i.e., DNA or RNA extraction, fatty acids, etc.), the evolution of this field has been enormous in recent years and is providing important insights into the relationships between the phylogeny and functionality of microbial communities. One of the major limitations to the progress of the metaproteomics approach for soil is the lack of a universal extraction protocol. More effort should be addressed to improve protein extraction methods while it is acknowledged that extensive soil heterogeneity in chemical, physical, and biological properties makes this task difficult. The first major distinction between the extraction methods proposed over the years is that between direct and indirect extractions. In the direct one, cell lysis occurs directly in the native soil sample. This guarantees a greater understanding of the interactions present in the rhizosphere but is more influenced by the matrix characteristics and is subject to the interfering substances co-extraction. The indirect method involves the physical separation of microbial cells from the soil and only after cell lysis and protein extraction. Although in a way the problem of interfering substances is solved, there is the risk of isolating only some microbial species, losing those strictly adhering to soil particles or that are non-culturable, and the whole pool of proteins released by the roots and microorganisms in the rhizosphere. Therefore, in order to fully exploit the enormous potential of the metaproteomics approach for soil, it is necessary to develop and use a type of direct and in-situ extraction. As seen in the literature, the various protocols used to date for cell lysis and direct and indirect extractions from the soil significantly affect the quality and quantity of extracted proteins. In the absence of an optimal protocol, suitable for all soil types, a strategy to solve the problem could be to combine several extractions, including the pool of extracted proteins, before subsequent analysis. Another important aspect to improve is the quantification of the extracted proteins. Several studies concluded that colorimetric methods are not valid, and thus alternative approaches (i.e., quantifying amino acids after hydrolysis) must be taken into consideration to quantify and normalize the amount of proteins to be used in downstream electrophoresis and mass-spectrometry analyses. Finally, improvement of the overall metaproteomic workflow, not only the protein extraction methods, but also protein/peptide separation, mass spectrometry approaches, and bioinformatics coupled to metagenomics, will provide a more complete picture of the microbially mediated processes taking place in soil, also including the identification of low abundant proteins which are of paramount importance for soil sustainability and bioremediation.

## Figures and Tables

**Table 1:** Use of the NoviPure Soil Protein kit, comparison with other extraction techniques, and the starting matrix.

Reference	Matrix	NoviPure Soil Protein Kit/Comparative Extraction Techniques
Hansen et al. [71]	Activated Sludge	Kuhn et al. [23] Barr et al. [45] optimized Chourey et al. [22] optimized NoviPure Soil Protein Kit
Butterfield et al. [73]	Grassland sub-root soil	NoviPure Soil Protein Kit + Amicon® Ultra-4 Centrifugal Filter Units (30 KDa)
Mattarozzi et al. [54]	Serpentine soil	Chourey et al. [46] Keiblinger et al. [16] NoviPure Soil Protein Kit
Hori et al. [74]	*Pinus contorta* litter	NoviPure Soil Protein Kit
Mandalakis et al. [19]	Agricultural surface soil	Phenol-based extraction Optimized Al3+ -based method NoviPure Soil Protein Kit
Cheng et al. [70]	Stony Corals	TRIzol Phenol-based extraction Trichloroacetic acid (TCA)-acetone; Glass bead-assisted extraction NoviPure Soil Protein Kit
Yao et al. [75]	Tropical Soil	NoviPure Soil Protein Kit modified by Butterfield et al. [73]
Bona et al. [76]	*Vitis vinifera* rhizosphere	NoviPure Soil Protein Kit
Zhou et al. [77]	Entisol	NoviPure Soil Protein Kit
Ouyang et al. [78]	Vegetable garden surface soil	NoviPure Soil Protein Kit
Mattarozzi et al. [72]	Rhizospheric maize soil	Benndorf et al. [21] NoviPure Soil Protein Kit

**Table 2:** Examples of possible application areas of metaproteomics approaches for soil.

Nutrient Cycle	Biomass Degradation	Soil Microbial Ecology	Plant-Microorganism Interactions	Assessment of Environmental State	Contamination, Bioremediation, and Soil Restoration
Bastida et al., (2016) [48]	Aylward et al., (2012) [86]	Bastida et al., (2016 a) [87]	Bao et al., (2014) [88]	Bastida et al., (2014) [47]	Bastida et al., (2015a,b) [89,90]
Bastida et al., (2019) [49]	Butterfield et al., (2016) [73]	Fernandez-Martinez et al., (2019) [91]	Bona et al., (2019) [76]	Bastida et al., (2018) [57]	Benndorf et al., (2009) [92]
Canizares et al., (2011) [93]	Hori et al., (2018) [74]	Festa et al., (2017) [94]	Knief et al., (2012) [95]	Chen et al., (2019) [96]	Chen et al., (2020) [97]
Chen et al., (2019) [96]	Keiblinger et al., (2012 b) [98]	Maron et al., (2008) [99]	Lin et al., (2013) [51]	Liu et al., (2017) [55]	Chourey et al., (2013) [100]
Orellana et al., (2019) [101]	Liu et al., (2015) [102]	Martinez-Alonso et al., (2019) [103]	Manikandan et al., (2017) [104]	Liu et al., (2019) [105]	Christensen et al., (2019) [106]
Starke et al., (2016) [107]	Schneider et al., (2012) [108]	Mattarozzi et al., (2020) [72]	Renu et al., (2019) [69]	Starke (2017) [109]	Guazzaroni et al., (2013) [110]
Tan et al., (2019) [111]	Zhang et al., (2018) [112]	Sekhon et al., (2016) [113]		Wang et al., (2017) [114]	Halter et al., (2011) [115]
Yao et al., (2018) [75]		Sidibè et al., (2016) [116]	Wu et al., (2011) [117]		Lechner et al., (2018) [118]
Zecchin et al., (2018) [119]		Williams et al., (2010) [120]	Zampieri et al., (2016) [11]		Lünsmann et al., (2016) [121]
					Mattarozzi et al., (2017) [54]
					Ouyang et al., (2019) [78]
					Singleton et al., (2003) [38]
					Sukul et al., (2017) [122]

## Supplementary Materials

The following are available online at https://www.mdpi.com/1422-0067/21/22/8455/s1.
