# Supplementary material for: Soil Metaproteomics for the Study of the Relationships Between Microorganisms and Plants: A Review of Extraction Protocols and Ecological Insights
Source: Int J Mol Sci. 2020 Nov 11;21(22):8455. doi: 10.3390/ijms21228455 (PMC7697097; doi:10.3390/ijms21228455)
Supplement: Supplementary file 1 [file ijms-21-08455-s001.pdf]

Reference	Physics cell lysis	Crucial aspects of the proposed extraction protocol	Advances	Disadvantages
Ogunseitan et al.(1993)	Boiling method<br><br>Freeze-thaw method	1:1 water and extraction buffer (100 mM TrisHCI pH 6.8, 200 mM dithiothreitol, 4% sodium dodecyl sulfate (SDS), 20% glycerol, and 0.2% bromophenol blue)<br><br>1:1 water and extraction buffer (50 mM Tris-HC1 (pH 7.6), 10% sucrose, 1 mM dithiothreitol (DTT)), lysozyme (600 ug / ml), EDTA (2 mM), 0.2% polyoxyethylene 20 cetyl ether	Both protocols allow direct extraction of extracellular and intracellular proteins	Reduced quantity of extracted proteins (1-5 µg/gboiling method, 20-50µg/g freeze method);<br><br>Co-extraction of humic substances
Criquet et al. (2002)	_	Comparison between different extraction protocols (distilled water, four salt solutions CaCl2, KCl, NaCl and Na2SO and five pH 6 buffers (succinate, bis-TRIS, PO3–4, pyrophosphate, citrate).<br>Protein concentration with dialysing membrane and PEG (15–20 kDa)	Extraction from forest litter<br>The protocol proposes strategies to limit the co-extraction of humic substances	No SDS-PAGE and mass spectrometry analysis performed
Murase et al. (2003)	_	67 mM phosphate buffer at various pH<br>precipitation with TCA<br>washes with ethanol and diethyl ether	The protocol showed that the increase in the extraction buffer pH corresponds to the increase in the co-extraction of interfering substances. The obtained pellet can be used for SDS-PAGE and subsequent sequencing.	Extraction limited to extracellular proteins only;<br><br>Large quantities of sample required;<br><br>SOM co-extraction
Singleton et al. (2003)	Liquid nitrogen<br><br>Bead beating	Ogunseitan et al.(1993) modified:<br>50 mm TRIS-HCl, 2 mm DTT, 4 mM EDTA, 0.1% Brij 58 (pH 7.6)	Cell lysis with liquid nitrogen allows to extract 32% more proteins than the “bead beating” method	Few protein bands detected in SDS-PAGE
Schulze et al. (2005)	_	Soil solution collected with suction plates and filtered with 0.2-µm acetate filter membrane, removal of DOM with sodium polytungstate, and 10% HF.	The proteins extracted from the solution sucked from the soil is rapid and compatible with proteomic analyzes	Extraction is limited to extracellular proteins isolated from DOM only
Benndorf et al.(2007)	_	Incubation in NaOH and phenolic extraction (Wang et al., 2003)	The use of phenol allows to remove DNA and RNA that negatively influence protein extraction	Phenol is a dangerous and difficult to handle compound<br>Co-extraction of humic acids
Masciandaro et al. (2008)	_	Chloroform fumigation-extraction method (Hofman and Dusek, 2003)<br>Comparison between different buffers:<br>0.1 m sodium pyrophosphate pH 7.0;<br>67 mM phosphate buffer (pH 6.0);<br>0.5 m potassium sulphate (pH 6.6).<br>PVPP and membranes to remove interfering substances	Intra and extracellular protein extraction<br>Correlation between the type of buffer used and the type and quantity of protein extracted	Co-extraction of humic substances that adversely effect on the SDS-PAGE<br>no identification of proteins extracted by mass spectrometry
Chen et al.(2009)	1h homogenization and incubation in the extractive buffers under stirring	“C-S-P-M method” 0.05 M citrate pH 8, SDS buffer (1% w / v SDS, 0.1M Tris-HCl, pH 6.8, 20mM DTT) followed by a classic phenol extraction (Wang et al.2003)	The combination of SDS buffer and phenolic extraction gave good results for the removal of the humic substance	Not applicable for deep proteome studies<br>reduced number of proteins extracted<br>low resolution of 2-DE separation
Chourey et al. (2010)	Boiling bath	Alkaline-SDS buffer (5% SDS, 50 mM Tris-HCl, pH 8.5; 0.15 M NaCl; 0.1 mM EDTA; 1 mM MgCl2; 50 mM Dithiothretiol (DTT)), Trichloroacetic acid (TCA)	Large number of extracted protein	Tested only on soils inoculated with bacteria

		overnight precipitation		
Wang et al. (2011)		"C/S-P-M method", the sequential citrate and SDS buffer extractions (Chen et al.,2009) is replaced by a single treatment of the two buffers	Decreased time required for extraction<br>Increased number of extracted proteins<br>Good degree of reproducibility	co-extraction of humic acids visible on the SDS PAGE
Keiblinger et al. (2012b)	_	Comparison between 4 extractive protocols<br>SDS buffer,<br>NaOH + phenol extraction,<br>SDS buffer + phenol extraction,<br>Washing steps, SDS buffer + phenol extraction	SDS buffer followed by extraction with phenol allowed the extraction of a greater number of proteins	Use of harmful substances (phenol)<br>co-extraction of interfering substances
Nicora et al. (2013)	_	Soil pre-treatment with amino acid mixture, desorption buffer, SDS-TCA acetone extraction	Compatible with protein extraction, tryptic digestion and mass spectrometric analysis<br>Increase protein identifications	The effectiveness of the protocol varies according to the type of soil
Quian et al. (2017)	_	Detergent based extraction buffer (SDS), precipitation in trichloroacetic acid (TCA) ,filtration at 10 kDa (pH 2 ~ 3)	Qualitative improvement of extracted proteins<br>protocol compatible with subsequent proteomic analysis<br>reduced co-extraction of interfering substances	Proteolytic peptides of microorganism mixture were added to the soil samples
Greenfield et al. (2018)	_	14C-labeled soluble proteins incubated in soil and then extracted with different solutions to evaluate the best combination	Good recovery with NaOH (0.1 M; 61–80%) and Na-pyrophosphate (0.05 M, pH 7.0; 45–75%)	Specific protocol for water-soluble proteins only
Callister et al.(2018)	Beads mix/Sonication	Folk et al.(1951) midified:<br>2:1 chloroform:methanol<br>SDS-Tris buffer<br>20% trichloroacetic	Suitable for 1D LC-MS/MS and 2D LC-MS/MS	Poor reproducibility<br>Laborius protocol
Mandalakis et al. (2018)	TissueLyser II (Qiagen, Venlo, Netherlands)	Protocol for DNA extraction readapted (Dong et al. 2006)<br>Flocculation of humic substances with trivalent aluminum ion Al^{3+}	Non-toxic, inexpensive and viable approach	Pellet of P. putida cells added to the soil sample
Renu et al. (2019)	Liquid nitrogen	Optimization of Keiblinger et al., 2012 and Benndorf et al., 2007 protocols.<br>Extraction buffer (1:1 (v/v) phenol (pH 8.0) and SDS phenol buffer (50 mM Tris, 1% SDS, pH 7.5) + PVPP	Good number of peptides identified by mass spectrometry	Use of harmful substances (phenol)<br>co-extraction of interfering substances<br><br>Poor quantity and quality of the bands obtained on SDS page

Table S1: Advances and disadvantages of the main extractive protocols
